# Regadenoson stress induced wall motion abnormalities during cardiac MRI

**DOI:** 10.1186/1532-429X-17-S1-P96

**Published:** 2015-02-03

**Authors:** Kalindi Parikh, Patricia W Bandettini, Marcus Y Chen, Jeannie H Yu, Sujata M Shanbhag, Andrew E Arai

**Affiliations:** National Heart, Lung and Blood Institute, National Institutes of Health, Bethesda, MD USA

## Background

Wall motion abnormalities are central to dobutamine stress CMR but have not been studied with regadenoson. This study was designed to compare the diagnostic performance of regadenoson regional wall motion abnormalities (RWMA) versus first-pass perfusion in the detection of significant coronary artery disease (CAD).

## Methods

Patients underwent regadenoson CMR that included 3 (basal, mid, apical) slices of rest and peak stress real-time cines, with matching stress and rest first-pass perfusion, and late gadolinium enhancement (LGE) imaging.

The reference standard for presence or absence of CAD was derived from invasive coronary angiography or coronary CT angiography (CTA). Invasive angiography established significant CAD based on a threshold of ≥70% stenosis and could rule-in or exclude CAD. CTA was only used to exclude CAD if the calcium score was <100 and no stenosis was >30%; CT was not used to diagnose CAD.

Two blinded, readers qualitatively scored RWM of pre and post-regadenoson.

## Results

26 of 49 patients had at least one 70% coronary artery stenosis. The sensitivity and specificity of regadenoson perfusion for detecting significant CAD was 92 and 91%, respectively. In comparison, the sensitivity and specificity of RWMA on stress imaging was 58 and 96%, respectively, and a new or worsening RWMA on stress compared to rest yielded a sensitivity and specificity of 38 and 100%, respectively. The sensitivity and specificity of LGE was 65 and 87%, respectively. Inter-reader agreement for RWMA was good (kappa 0.63).

## Conclusions

Although sensitivity is poor, a regadenoson-induced wall motion abnormality seen on CMR likely indicates significant CAD.

## Funding

Funded by the Intramural Research Program of the National Heart, Lung, and Blood Institute (NHLBI), National Institutes of Health, Bethesda, MD.

**Table 1:** Comparison of diagnostic accuracy of differing stress CMR exam components

	Sensitivity	Specificity	Accuracy	PPV	NPV
Perfusion	0.92	0.91	0.92	0.92	0.91
Stress RWMA	0.58	0.96	0.76	0.94	0.67
Induced RWMA	0.42	1.00	0.69	1.00	0.61
LGE	0.65	0.87	0.76	0.85	0.69

PPV= positive predictive value; NPV= negative predictive value; RWMA= regional wall motion abnormality; LGE= late gadolinium enhancement

